# 

**Published:** 1903-10-10

**Authors:** 


### CONTENTS.

**The Science and the Art of Feeding** - - - - - 25

**Roman Catholic Inebriates and their Religion** - 26

**Annotations** - - - - - - - - - - - - - 27

**Notes and News** - - - - - - - - - - - - 28

**Hospital Clinics and Medical Progress—**

Enlarged Tonsils - - - - - - - - - - - - 29

Chronic Nephritis Without Albuminuria - - - - - - 29

Treatment of Addison's Disease - - - - - - - - 30

The Value of Blood Examinations in Appendicitis - - - 30

Pathology of Gall-Stones - - - - - - - - - 31

Progress in Surgery of the Stomach - - - - - - 32

Progress in Phototherapy and Electro-therapeutics - 33

Progress in Nerve Surgery - - - - - - - - - 34

**New Appliances and Things Medical** - - - 35

**The Book World of Medicine and Science** - - 36

**Hospital Administration—**

British Institutions for the Care of the Inebriate.—II. - - - 37

Rise and Growth of Vaccination Law.—II. - - - - - 38

Improvements at Charing Cross Hospital - - - - - 39

Glances at the Hospitals - - - - - - - - - 39

**The Student of Medicine** - - - - - - - 40

*NURSING SECTION.*

**Notes on News from the Nursing World—**

Nurses Needed in European Turkey—Queen Alexandra's Imperial Military Nursing Service—Lady Dudley's Nursing Scheme—The Benefit of English Training—District Nurses in the Canton of Berne—Guardians on the Marriage of Nurses—The New Matron of Bethnal Green Infirmary—Nursing and Physical Strength—A Lady Doctor as Superintendent Nurse—A Victorian Nurse in Fort Frances—Guardians and Queen's Nurses in Sheffield—An Incident at an Isolation Hospital—The Workmen's Nurse and her Badge—Nurses and Swimming—A Nurses' Home for Smethwick—A Flourishing Village Nursing Association—Funeral of a Nurse at York—The Pension Fund's Bonus Additions—The "Handy Woman" not Extinct—Plaistow District Nurses - - - 23-26

**The Examination of the Urine** - - - - - - 27

**Hints on How to Start a District Nursing Association** - - - - - - - - - - - - 28

**An Impression from the Land of the Shamrock** 29

**The Victorian Trained Nurses' Association** - 31

**The International Hospital in Venice** - - - 31

**Bulbs for Hospital Decoration** - - - - - - 32

**For Elderly Nurses** - - - - - - - - - - 32

**Everybody's Opinion** - - - - - - - - - 33

**Novelties for Nurses** - - - - - - - - - 34

**Appointments** - - - - - - - - - - - 34

**The Nurses' Bookshelf** - - - - - - - - - 34

**Echoes from the Outside World** - - - - - - 35

**For Reading to the Sick** - - - - - - - 36

**Notes and Queries** - - - - - - - - - - 35

